# Emotional engagement as a mediator in design thinking-based entrepreneurship education: a control-value theory perspective on innovation and interpersonal competency

**DOI:** 10.3389/fpsyg.2026.1846895

**Published:** 2026-07-07

**Authors:** Zhipeng Ye, Eng Hoe Wee, Jiajian Wang, Guimei Zhang, Dake Lin

**Affiliations:** 1 School of Economics and Management, Sanming University, Sanming, China; 2 Faculty of Education, Languages, Psychology and Music, SEGi University, Petaling Jaya, Selangor, Malaysia; 3 School of Education and Music, Sanming University, Sanming, China; 4 Logistics Department, Wenzhou University, Wenzhou, China

**Keywords:** achievement emotions, affective attitude, control-value theory, design thinking, entrepreneurship education, innovation competency, interpersonal competency

## Abstract

**Introduction:**

Design Thinking (DT) has been increasingly adopted in entrepreneurship education (EE), yet the psychological mechanisms through which DT contributes to entrepreneurial competency development remain insufficiently understood. Drawing on Control-Value Theory (CVT) as an interpretive framework, this study examines whether and how affective processes are associated with the development of entrepreneurial competencies in a quasi-experimental educational context.

**Methods:**

A pre-test/post-test design with non-randomly assigned intact classes was employed with 198 undergraduate students (a predominantly female, second-year cohort) from a Chinese university; the same instructor taught both the Design Thinking Teaching Method (DTTM) and Conventional Teaching Method (CTM) groups (a within-instructor design), and pre-test scores were included as covariates. Using Partial Least Squares Structural Equation Modeling (PLS-SEM), the study investigated the role of Affective Attitude (AC) as a potential mediator linking DT pedagogy with Innovation Competency (INC) and Interpersonal Competency (IC).

**Results:**

The results showed that DT was significantly associated with higher AC compared with conventional instruction. Furthermore, AC fully mediated the relationship between DT and INC, suggesting that affective engagement may represent an important pathway through which DT relates to INC development. For IC, the findings revealed an exploratory competitive mediation pattern, in which DT was associated with a negative direct effect on IC alongside a positive indirect association through AC. Given that IC was measured using a two-item scale and cultural orientations were not directly assessed, this pattern should be interpreted cautiously as a tentative finding rather than a confirmed mechanism.

**Discussion:**

The study contributes to EE research by providing a CVT-informed explanation of how affective processes may accompany competency development in DT-based learning environments, moving beyond simple outcome comparisons. The findings highlight the importance of considering students' emotional engagement when designing experiential EE and suggest that future research should directly measure control and value appraisals to further test the proposed CVT-based mechanism.

## 1 Introduction

In the rapidly evolving landscape of higher education, Entrepreneurship Education (EE) has transitioned from a niche discipline focused on business planning to a transversal competency framework essential for all students (Neck and Greene, 2011). This shift is particularly pronounced in China, where the continuous universalization of higher education and intense employment pressures have made entrepreneurial competencies an essential competency set for graduates' career development (Li et al., 2016). Entrepreneurial competencies in this context are increasingly understood as a multidimensional capability set rather than a single technical skill. They typically encompass creativity and innovation, opportunity recognition, initiative, problem-solving, interpersonal communication, teamwork, networking, and adaptability, all of which enable students to generate novel ideas, collaborate effectively, and translate ideas into actionable entrepreneurial practice (Man et al., 2002; Mitchelmore and Rowley, 2010; Morris et al., 2013; Sánchez, 2013). Within this pedagogical shift, Design Thinking (DT) has emerged as one of the indispensable methodological approaches. Unlike traditional predictive logic that emphasizes market analysis, memorization, and financial forecasting, DT advocates for a creation-oriented logic rooted in empathy, iteration, and user-centricity (Liedtka, 2015). This methodological pivot promises not only to equip students with functional skills but to fundamentally reshape their cognitive and affective relationship with entrepreneurship, making it highly relevant for students from non-business backgrounds who may initially perceive entrepreneurship as daunting (Linton and Klinton, 2019).

Despite the widespread adoption of DT, empirical evidence regarding its efficacy remains paradoxical. While some studies report significant gains in students' entrepreneurial competencies (Liedtka, 2015; Nabi et al., 2017), others suggest that DT interventions yield negligible differences compared to traditional methods when measured strictly by outcome-based scores (Linton and Klinton, 2019). This inconsistency points to a critical methodological limitation in the current literature: the predominance of “black box” assessments. By focusing primarily on whether DT outperforms traditional methods (mean comparison), researchers often overlook how and why it works. Specifically, the mechanisms through which experiential learning translates into competency acquisition remain underexplored (Lackéus, 2015). If mere exposure to DT tools does not guarantee skill improvement, there must be a psychological mediator that activates the learning process, a driver that traditional outcome-based evaluations fail to capture (Pekrun, 2006).

This study posits that the missing link lies in the affective domain. According to the Control-Value Theory of Achievement Emotions (CVT), emotions are not merely byproducts of learning but active antecedents of cognitive performance (Pekrun, 2006). The theory proposes that achievement emotions arise from two fundamental cognitive appraisals: control appraisals, referring to students' perceived influence over learning processes and outcomes, and value appraisals, concerning the subjective importance of the task or result (Pekrun et al., 2007). These dual appraisal dimensions interact to generate specific emotional states, which in turn influence learning strategies, cognitive resources, and ultimately academic performance. In the Chinese higher education context, students' entrepreneurial competency development is shaped not only by cognitive resources but also by the affective conditions of the learning environment. Research has consistently shown that China's examination-oriented educational culture fosters heightened sensitivity to failure and evaluative threat (Fang et al., 2023; Zhao et al., 2010), which, through the lens of CVT, constitutes a low-control, high-value appraisal configuration that generates anxiety and inhibits exploratory learning (Pekrun, 2006). For these students, the primary barrier to competency acquisition may therefore be less a deficit of intellect than a deficit of psychological safety and emotional engagement (Edmondson, 1999; Lackéus, 2015). We argue that the true pedagogical value of DT lies in its capacity to function as an Affective Catalyst for competency development. By this term we mean a pedagogical condition that, by creating learning conditions theoretically consistent with enhanced control and value appraisals, raises positive affective engagement (operationalized in this study as AC) to a level at which it becomes the pathway through which the methodological tools of DT translate into competency gains. Specifically, by normalizing failure through iterative prototyping and grounding tasks in authentic human needs, DT may create learning conditions that, from a CVT perspective, are theoretically associated with enhanced control and value appraisals and thus with the positive activating emotions that CVT identifies as prerequisites for deep learning (Pekrun, 2006; Simonton and Garn, 2020).

CVT may also help to interpret features of the Chinese educational context in which this study was conducted. In Chinese society, especially within the educational domain, academic achievement is often emphasized as an important pathway for personal advancement and bringing honor to one's family (Xie and Liu, 2025). Within this cultural environment, the construct of “value appraisal” acquires a distinctly expanded meaning. CVT distinguishes between intrinsic value, derived from the inherent enjoyment of a learning activity, and extrinsic value, associated with the instrumental utility of the activity for achieving desired outcomes (Pekrun, 2006). In the Chinese context, extrinsic value is powerfully amplified by Confucian norms of filial piety, in which academic success serves as a primary vehicle for honoring one's family and fulfilling intergenerational obligations (Chen and Ho, 2012). Students' value appraisals are therefore shaped not merely by personal interest but by a dense web of family expectations, social evaluation, and anticipated socioeconomic mobility (Fang et al., 2023; Xie and Liu, 2025). A critical consequence of this culturally reinforced extrinsic value is that even learning tasks perceived as tedious may carry disproportionately high subjective importance, creating what CVT would characterize as a high-value condition. When this elevated value is accompanied by uncertainty about one's ability to meet such expectations, the resulting appraisal configuration is precisely the one CVT predicts will generate anxiety and evaluative tension (Pekrun, 2006; Shao et al., 2020).

The experience of “control appraisal” presents a complementary set of context-related characteristics. China's educational system is typically marked by high structural uniformity, standardized evaluation criteria, and limited curricular choice at the student level (Li et al., 2016), features that may constrain students' perceived autonomous control over learning processes and outcomes. However, this reduced macro-level autonomy does not necessarily translate into perceived helplessness. Research suggests that Chinese students can develop a sense of effort-based control, a belief that sustained diligence can compensate for structural constraints and enable them to meet the high standards set by society and family (Liu et al., 2025). This represents a culturally distinct pathway to perceived control that differs from the agency-based control more commonly emphasized in Western educational settings.

To deconstruct this mechanism, this research integrates a quasi-experimental design with Partial Least Squares Structural Equation Modeling (PLS-SEM). Unlike traditional variance analyses (ANOVA/*t*-tests) that treat groups as monolithic blocks, PLS-SEM allows for the modeling of the hypothesized pathways while adjusting for measured baseline differences (Hair et al., 2019). This approach enables us to isolate the specific contribution of Affective Attitude (AC) to competency development. Furthermore, this study uniquely investigates whether this affective pathway functions as a compensatory mechanism, particularly exploring the complex competitive mediation phenomena (Zhao et al., 2010) that may arise when the demanding, critique-intensive collaboration central to DT places strain on students' interpersonal experience. We treat any cultural dimension of this pattern as a tentative interpretation rather than a tested premise, as cultural variables were not measured in this study. By shifting the focus from “scores” to “drivers,” this study offers a nuanced explanation for the mixed results in previous literature and provides empirical evidence for the psychological power of DT.

### 1.1 Control-value theory of achievement emotions

The CVT of Achievement Emotions provides the primary theoretical framework for this study (Pekrun, 2006; Pekrun et al., 2007). CVT posits that achievement emotions are determined by two classes of cognitive appraisals: control appraisals (subjective beliefs about one's ability to influence learning processes and outcomes) and value appraisals (the perceived importance of the learning task or its outcomes). The interaction of these dimensions generates predictable emotional patterns: high control paired with high value produces positive activating emotions (e.g., enjoyment, hope), whereas high value with low control generates anxiety or hopelessness (Pekrun, 2006). Critically, CVT treats these emotions not as static traits but as dynamic, situationally responsive states that fluctuate as learning contexts change (Pekrun, 2024). This dynamic quality makes CVT particularly suited to analyzing pedagogical interventions that fundamentally alter the learning environment.

CVT further specifies that positive activating emotions enhance learning outcomes through multiple pathways, including broadened attentional scope, increased cognitive flexibility, deeper processing strategies, and stronger intrinsic motivation (Pekrun, 2006; Simonton and Garn, 2020). Empirical studies have confirmed these pathways in diverse educational contexts, including university physical education (Garn et al., 2017), foreign language learning in China (Li, 2021), and science education (Chen et al., 2020). In the Chinese educational context, where academic achievement is deeply intertwined with family expectations and social evaluation (Xie and Liu, 2025), and where students often exhibit heightened sensitivity to failure (Fang et al., 2023), CVT's emphasis on perceived control as a buffer against negative emotions is especially relevant. DT's iterative, failure-tolerant pedagogy may be particularly effective at modifying the appraisal configurations that CVT identifies as anxiety-producing.

Importantly for the present study, CVT recognizes that achievement emotions operate at multiple levels of temporal granularity. At the situational level, discrete emotions such as enjoyment or anxiety arise in response to specific learning episodes; at the dispositional level, these recurring emotional experiences aggregate over time into stable affective patterns that characterize a student's typical emotional orientation toward a given domain (Goetz et al., 2006; Pekrun, 2006). Pekrun (2024) further elaborated this hierarchical structure in the generalized CVT, demonstrating that domain-specific affective dispositions emerge from the cumulative history of situational appraisal-emotion episodes within a particular learning context. This theoretical distinction between situational emotions and accumulated affective dispositions is critical for understanding how a sustained pedagogical intervention, such as a 12-week DT-based EE curriculum, can generate measurable shifts not only in momentary emotional states but in students' broader affective orientation toward the learning domain. It should be noted at the outset that the present study operationalizes and tests only the latter segment of this chain, that is, the link from achievement emotion to learning outcome. The antecedent appraisal stage, namely control and value appraisals, is drawn on as theoretical rationale but was not directly measured. CVT therefore functions here as an interpretive framework for the emotion to competency relationship rather than as a full test of the appraisal to emotion to outcome sequence, a boundary we revisit when interpreting the AC measure in Section 1.2 and in the limitations.

### 1.2 Design thinking and affective attitude

The DT pedagogical approach creates learning environments with structural features that, according to CVT's logic, should systematically enhance students' control and value appraisals. DT's iterative, failure-tolerant design philosophy normalizes errors as learning opportunities rather than threats, thereby increasing students' perceived control over outcomes (Linton and Klinton, 2019). Simultaneously, DT's empathy phase grounds learning in real-world user needs, enhancing intrinsic task value (Liedtka, 2015). According to CVT, this combination of enhanced control and value should generate positive activating emotions, particularly enjoyment and engagement (Simonton and Garn, 2020). Empirical research supports this reasoning: Li (2026) demonstrated that uncertainty-enhanced inquiry tasks improved students' control beliefs and enjoyment, and Forsblom et al. (2021) found that perceived classroom support longitudinally predicted positive achievement emotions.

Building on CVT's hierarchical model of emotional experience, we operationalize the cumulative affective outcome of these repeated appraisal processes as AC. CVT distinguishes between state-level achievement emotions, which arise from momentary appraisals during specific learning episodes, and trait-level affective dispositions, which represent the habitualized patterns of emotional responding that accumulate through repeated exposure to a given learning domain (Goetz et al., 2006; Pekrun, 2006). Over the course of a sustained 12-week DT intervention, students experience numerous iterative cycles of empathizing, ideating, prototyping, and testing, each of which constitutes a discrete appraisal-emotion episode. According to CVT's reciprocal causation principle, these repeated experiences of enhanced control (through normalized failure) and enhanced value (through authentic user engagement) are expected to consolidate into a stable, domain-specific affective disposition (Pekrun, 2006; Pekrun and Stephens, 2010). It is this accumulated dispositional pattern, rather than any single momentary emotion, that AC is designed to capture.

Specifically, AC is measured using the three-item affective subscale of the Undergraduate Attitude toward Entrepreneurship Education Scale (UAEES) adapted for the Chinese context by Zhang et al. (2022), which assesses students' affective satisfaction, positive engagement, and psychological investment directed toward the entrepreneurship learning experience. We acknowledge that AC, as an attitudinal measure, is conceptually broader than the discrete achievement emotions (e.g., enjoyment, hope, pride) specified by CVT's taxonomy. However, we argue that this operationalization is theoretically justified on two grounds. First, CVT itself posits that the aggregate affective experience across a sustained learning period reflects the dispositional endpoint of repeated appraisal-emotion cycles (Pekrun, 2006, 2024), and AC captures precisely this aggregate pattern. Second, the UAEES affective subscale items (e.g., “I am happy to have had entrepreneurship education in my university”) directly tap the experiential signatures of the positive activating emotions (enjoyment, enthusiasm, engagement) that CVT predicts will result from high-control, high-value appraisal configurations (Pekrun et al., 2007; Simonton and Garn, 2020). While the scale does not differentiate among specific emotion categories, its content aligns with the functional outcomes CVT attributes to positive activating emotions as a class. We nevertheless acknowledge this operationalization as a limitation: AC cannot capture the differential contributions of distinct emotions (e.g., enjoyment vs. hope vs. pride), nor does it directly assess the appraisal dimensions (control and value) that CVT posits as their antecedents. We return to these measurement boundaries in Section 4.5.

Based on CVT's appraisal-emotion logic, we hypothesize:

**H1**: The Design Thinking Teaching Method (DTTM) significantly and positively enhances students' Affective Attitude (AC) compared to the Conventional Teaching Method (CTM).

### 1.3 Affective attitude and entrepreneurial competency development

CVT predicts that positive activating emotions facilitate cognitive performance by broadening attentional resources and promoting flexible, creative processing (Pekrun, 2006). For Innovation Competency (INC), this prediction is particularly compelling because innovation requires divergent thinking, risk tolerance, and creative problem-solving. From a CVT perspective, these cognitive capacities are specifically those that positive activating emotions (e.g., enjoyment, hope) are theorized to enhance through broadened attention and increased willingness to explore novel approaches (Pekrun, 2006; Simonton and Garn, 2020). This reasoning finds complementary support in dual-process theory, which characterizes innovation as a form of “hot cognition” that depends on emotional engagement rather than purely rational information processing (Gladwin and Figner, 2014; Tinajero et al., 2024). Empirical evidence supports this link: Su et al. (2022) found that positive affect mediated the relationship between organizational support and creative performance, and Ovbiagbonhia et al. (2019) demonstrated that perceived learning environment quality predicted students' INC. This emphasis on the cognitive benefits of positive affect is consistent with broaden-and-build accounts of how positive emotions widen attention and thought-action repertoires (Fredrickson, 2001) and with evidence that positive affect supports creativity at work (Amabile et al., 2005).

For Interpersonal Competency (IC), CVT suggests that positive emotions reduce defensive reactions and promote social openness and perspective-taking (Pekrun, 2006). Within CVT's framework, positive affect serves as the psychological precondition that lowers interpersonal defensiveness and increases willingness to engage with others (Pekrun, 2006). Complementing this precondition logic, social learning theory (Bandura, 1991) specifies the process mechanisms through which interpersonal skills are actually acquired once these affective preconditions are met, including behavioral modeling and situated practice in collaborative settings. Research on collaborative learning has shown that positive emotional states predict more constructive peer interactions and deeper engagement in group tasks (Destacamento, 2018). Based on this reasoning:

**H2**: Affective Attitude (AC) has a significant positive impact on Innovation Competency (INC).

**H3**: Affective Attitude (AC) has a significant positive impact on Interpersonal Competency (IC).

### 1.4 The mediating role of affective attitude

Integrating the above arguments, we propose that AC mediates the relationship between pedagogical method and competency outcomes. CVT's emphasis on emotions as proximal antecedents of learning outcomes suggests that pedagogical interventions influence competency development substantially through affective pathways, not solely through direct skill transfer. Prior research in EE has similarly argued that affective engagement is a critical but underexplored mediator between educational inputs and competency outputs (Lackéus, 2015; Nabi et al., 2017).

For INC, we expect that DT's effect operates primarily, though not necessarily exclusively, through AC. DT tools such as brainstorming and prototyping are methodological guidelines that, in isolation, may be insufficient to overcome the deeply ingrained failure-avoidant patterns of students accustomed to high-stakes educational cultures (Fang et al., 2023). Affective activation is hypothesized to be the necessary catalyst that enables students to use these tools effectively for creative exploration. We therefore predict that the indirect pathway through AC will constitute the dominant mechanism, while acknowledging that a residual direct effect of DT on INC may also exist if the methodological tools themselves contribute to skill acquisition independently of affective engagement.

For IC, we hypothesize a more complex pattern. DT's intensive collaborative component requires students to critique peers' ideas and manage conflicting viewpoints in ways that may violate Chinese cultural norms of harmony and face preservation (King and Wei, 2018). Drawing on Pekrun's (2024) recent extension of CVT to social emotions, we propose that the control-value appraisal framework can be applied to this interpersonal sub-domain as a theoretical extension. Specifically, while DT enhances the overall value of the task through its empathy phase, intensive collaboration simultaneously reduces students' perceived control in the interpersonal domain: they cannot predict how candid criticism will affect peer relationships. According to CVT, this high-value/low-control configuration generates anxiety and defensive behavior (Heckhausen et al., 2010; Pekrun, 2006), which may manifest as reduced self-reported IC (Vu et al., 2021). However, when DT successfully activates a positive overall AC, this affective engagement may buffer against domain-specific interpersonal anxiety through cognitive reappraisal, that is, reinterpreting friction as constructive feedback (Albarracin et al., 2024; Balzarotti et al., 2017). This dual-pathway reasoning leads us to expect a competitive mediation pattern (Zhao et al., 2010), where the direct and indirect effects have opposite signs.

We note that this prediction is theoretically contingent on cultural contexts characterized by high face concern and collectivist harmony orientation (King and Wei, 2018). In educational settings where candid peer critique is normatively expected rather than culturally threatening, the negative direct effect may be attenuated or absent. This cultural boundary condition should be directly tested in future cross-cultural research. We emphasize that these cultural constructs (e.g., face concern and harmony orientation) frame our theoretical reasoning but were not measured in the present study; they therefore inform our interpretation rather than serving as tested variables.

Based on this reasoning, we hypothesize:

**H4**: Affective Attitude (AC) mediates the relationship between the pedagogical intervention (DTTM vs. CTM) and Innovation Competency (INC).

**H5**: Affective Attitude (AC) mediates the relationship between the pedagogical intervention (DTTM vs. CTM) and Interpersonal Competency (IC).

**Figure 1:**
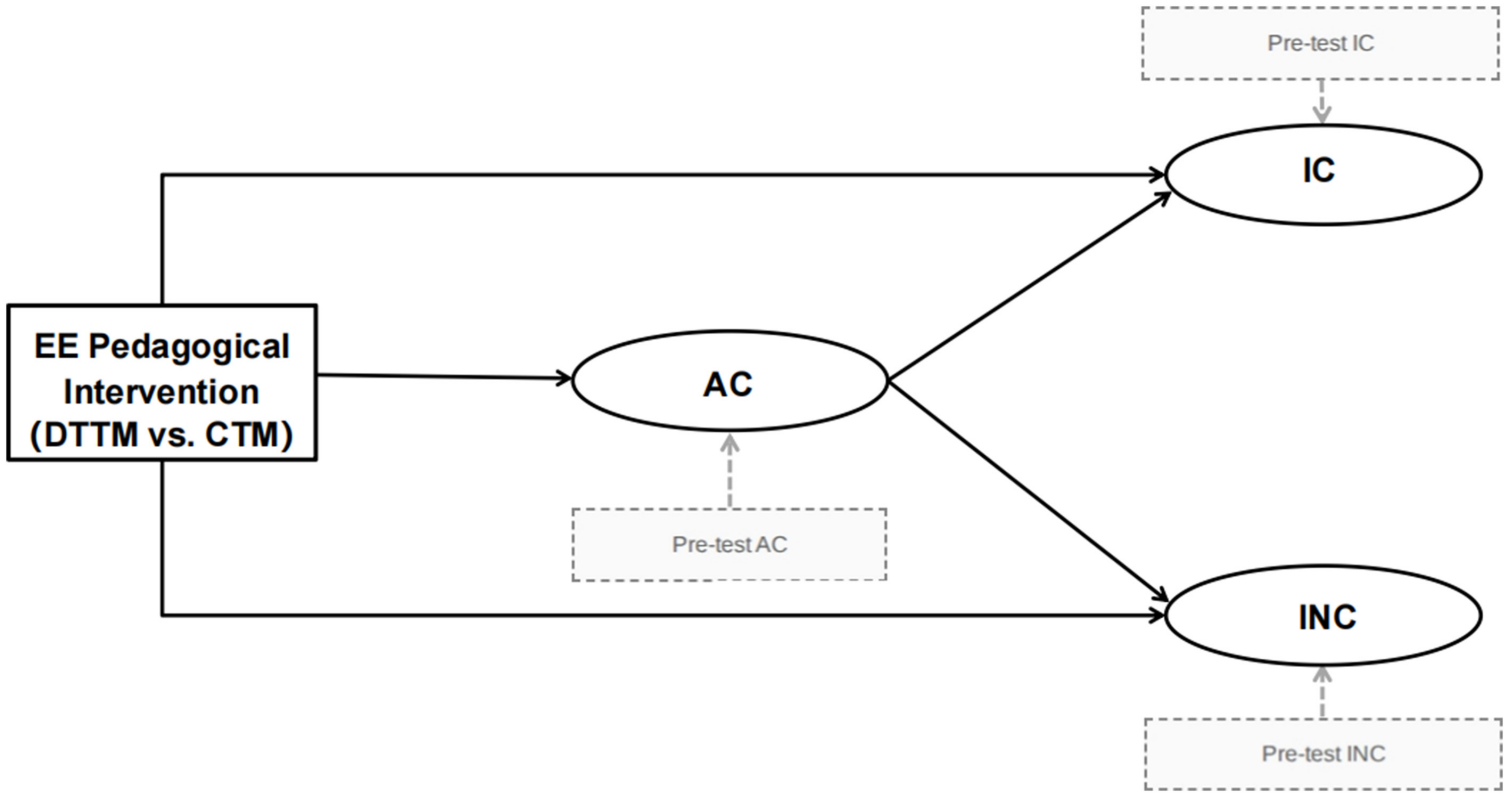
Conceptual framework. EE, Entrepreneurship Education; CTM, Conventional Teaching Method; DTTM, Design Thinking Teaching Method; AC, Affective Attitude; IC, Interpersonal Competency; INC, Innovation Competency.

The conceptual model is presented in Figure 1. Pre-test scores for all variables are included as control variables to isolate developmental effects.

## 2 Materials and methods

### 2.1 Study design and participants

This study employed a quasi-experimental, pre-test/post-test design to investigate the underlying affective mechanisms driving entrepreneurial competency development. To isolate the pedagogical effect and control for confounding variables related to teaching styles, a “within-instructor design” was adopted (Charness et al., 2012). The same instructor delivered both the conventional and experimental curricula concurrently (Deslauriers et al., 2019).

The two groups were constituted from pre-existing parallel classes arranged according to the university's original teaching schedule, rather than through random assignment or student self-selection. This naturalistic class allocation, a defining feature of quasi-experimental designs in intact educational settings (Anderson-Cook, 2005), reduces voluntary selection bias while preserving ecological validity. The final valid sample comprised 198 students, with the Experimental Group (DTTM, *n* = 99) and Control Group (CTM, *n* = 99). The sample was predominantly female (73.7%) and in their sophomore year (98.5%), with an average age of 19 years. Prior to data collection, institutional ethical approval was obtained from the Ethics Committee of SEGi University (Approval No. SEGiEC/SR/FOELPM/265/2024-2025). All participants provided written informed consent, which included information about the study's purpose, procedures, data confidentiality measures, and the right to withdraw at any time without penalty. The baseline equivalence of the two groups was confirmed through preliminary analyses (see Section 3.1).

#### 2.1.1 Statistical power analysis

An a priori statistical power analysis was conducted using G^*^Power 3.1 (Faul et al., 2009) to determine the minimum required sample size. Based on the PLS-SEM model specification (maximum number of arrows pointing at a single construct = 3), with a medium effect size (*f* ^2^ = 0.15; Cohen, 1988), a significance level of α = 0.05, and a target statistical power of 0.80, the minimum required sample size was calculated as 77. The actual sample size of 198 substantially exceeded this threshold, yielding an estimated *post-hoc* statistical power of 0.99, which indicates sufficient power to detect the hypothesized effects (Hair et al., 2019).

### 2.2 Procedure and intervention

The intervention spanned 12 weeks, comprising 36 instructional hours delivered in weekly 135-min sessions.

**Control Condition (CTM)**: The control group received the Conventional Teaching Method, focusing on theoretical knowledge transfer, textbook learning, and the traditional formulation of business plans.

**Experimental Condition (DTTM)**: The experimental group utilized the Design Thinking Teaching Method, structured around the five-stage framework (Empathize, Define, Ideate, Prototype, Test). Emphasizing a user-centric creation logic, the curriculum integrated empathy mapping, rapid prototyping, and stakeholder feedback.

To ensure high intervention fidelity, multiple procedural safeguards were implemented. First, both groups followed standardized curricula with clearly documented learning objectives, teaching schedules, and assessment criteria, ensuring that the instructional content was delivered consistently according to the research protocol. Second, the instructor maintained detailed teaching logs throughout the 12-week intervention period, recording session content, deviations from the planned curriculum, and observations about student engagement, which were reviewed weekly by the research team. Third, the experimental group received a physical DT manual and co-designed PowerPoint slides integrating DT concepts, which were not provided to the control group, thereby creating a clear and observable differentiation in instructional materials. Fourth, the instructor underwent a structured 1-month training program prior to the intervention, during which the research team collaboratively developed DT-integrated teaching materials and conducted practice sessions to ensure consistent and faithful implementation of the DT methodology (Charness et al., 2012). Together, these multi-layered procedural safeguards, including standardized curricula, teaching logs, differentiated materials, and systematic instructor training, provide converging evidence of high intervention fidelity, ensuring that the two groups received distinctly different pedagogical treatments as intended.

### 2.3 Measures

All items were measured on a 5-point Likert scale ranging from 1 (strongly disagree) to 5 (strongly agree). To rigorously isolate the developmental impact, pre-test scores for all variables were included as control variables in the analytical model. To reduce common method bias (CMB), the survey was administered anonymously, and items across different constructs were interspersed in the questionnaire (Podsakoff et al., 2003).

**Affective Attitude (AC)**: Measured using a 3-item subscale from the UAEES adapted for the Chinese context by Zhang et al. (2022). Sample item: “I am happy to have had entrepreneurship education in my university” (pre-test α = 0.66, post-test α = 0.75). Note: While pre-test α fell below the conventional 0.70 threshold, Cronbach's α is sensitive to the number of items, and composite reliability (CR) values exceeded 0.80 for all constructs (see Section 3.2), which is the recommended reliability indicator for PLS-SEM (Dijkstra and Henseler, 2015; Hair et al., 2019).

**Innovation Competency (INC)**: Measured using a 3-item subscale from the UECS revised by Wang (2023). Sample item: “I am willing to try to achieve the goal in different ways.” (pre-test α = 0.67, post-test α = 0.74).

**Interpersonal Competency (IC)**: Measured using a 2-item subscale from the UECS (Wang, 2023). Sample item: “I am good at building good relationships with others.” (pre-test α = 0.76, post-test α = 0.69). The 2-item scale represents a limitation of this study; future research should expand this measure to improve content coverage and reliability stability.

### 2.4 Data analysis strategy

PLS-SEM using SmartPLS 4 software was utilized. We selected PLS-SEM for three study-specific reasons rather than as a default option. First, because the primary objective was not to confirm an established covariance structure but to explore and predict competency development pathways in an educational intervention context, a variance-based approach was preferred; this aim, namely to identify the affective drivers of competency and to maximize the explained variance in the outcome constructs (reported via *Q*^2^), aligns with the variance-based logic of PLS-SEM rather than the global model-fit logic of covariance-based SEM. Second, IC is a two-indicator reflective construct, and two-indicator latent variables are prone to identification problems in covariance-based SEM, whereas PLS-SEM accommodates them; consistent with this, our consistent PLS robustness check, which most closely mirrors covariance-based estimation, returned an inadmissible solution for this construct (Section 3.4), indicating that covariance-based estimation is unstable in the present case. Third, the data are non-normal Likert responses with a dummy-coded exogenous variable, conditions under which PLS-SEM remains robust (Hair et al., 2019; Henseler et al., 2015). We further acknowledge that covariance-based SEM with robust estimators is a reasonable alternative for the larger constructs, and we note that our substantive conclusions about INC do not depend on the choice of estimator. We coded the Pedagogical Intervention as a dummy variable (0 = CTM, 1 = DTTM). Throughout this paper, including all tables and figures, the construct labels AC, INC, and IC denote the post-test (outcome) measures unless explicitly prefixed with “Pre-test” (or “Pre-”); the corresponding pre-test scores (Pre-AC, Pre-INC, Pre-IC) were entered as control variables predicting their respective post-test counterparts to isolate the developmental effect of the intervention. Mediation analysis followed the rigorous bootstrapping procedures (5,000 resamples) and typologies outlined by Zhao et al. (2010). We use the term mediation in its statistical sense. Because participants were not randomly assigned, a causal mediation interpretation would require assumptions of no unmeasured confounding of the intervention to mediator and mediator to outcome relations that a quasi-experiment cannot fully guarantee, so the mediation results are interpreted as associational pathways.

The measurement model was assessed following established PLS-SEM guidelines, including indicator reliability (outer loadings > 0.708), internal consistency reliability (CR > 0.70; rho_A), convergent validity (Average Variance Extracted (AVE) > 0.50), and discriminant validity (Fornell-Larcker criterion and Heterotrait-Monotrait (HTMT) ratio < 0.85; Henseler et al., 2015). The structural model was evaluated using path coefficients, coefficient of determination (*R*^2^), effect sizes (*f* ^2^), and predictive relevance (*Q*^2^) via blindfolding (*d* = 7).

## 3 Results

### 3.1 Preliminary analysis

Prior to the main analysis, baseline equivalence between the experimental and control groups was verified. As shown in Table 1, no statistically significant differences were found between the two groups on any pre-test variable (all *p* > 0.05) or demographic characteristics, confirming that the groups were comparable at baseline.

**Table 1:** Baseline equivalence between experimental and control groups.

Variable	DTTM (M ±SD)	CTM (M ±SD)	Test statistic	*p*	Result
Pre-AC	3.38 ± 0.63	3.49 ± 0.62	*U* = 4625.50	0.213	n.s.
Pre-INC	3.47 ± 0.63	3.59 ± 0.59	*U* = 4558.00	0.165	n.s.
Pre-IC	3.39 ± 0.75	3.49 ± 0.64	*U* = 4701.00	0.321	n.s.
Gender (%F)	Female 70.7%	Female 76.8%	χ^2^ = 0.652	0.419	n.s.
Age (M ± SD)	19.34 ± 0.78	19.40 ± 0.75	*t* = −0.554	0.580	n.s.
Grade (%F)	Sophomore 98.0%	Sophomore 99.0%	χ^2^ = 1.005	0.605	n.s.

n.s., not significant (*p* > 0.05). Mann-Whitney *U* tests used for ordinal pre-test variables; χ^2^ for gender and grade; independent-samples *t*-test for age.

Initial comparisons using Quade's Non-parametric ANCOVA revealed that DTTM significantly enhanced students' overall AC compared to CTM (*F* = 6.17, *p* = 0.014). However, the intervention did not yield a statistically significant direct advantage in INC (*F* = 0.05, *p* = 0.823) and was outperformed by CTM in IC (*F* = 8.38, *p* = 0.004). These mixed outcome-based results necessitated the PLS-SEM analysis to unearth the underlying psychological drivers.

#### 3.1.1 Common method bias assessment

Although procedural remedies such as temporal separation (pre-test and post-test data collection) and psychological separation (interspersing items) were implemented to mitigate common method variance (Podsakoff et al., 2003), statistical assessments were further conducted to rigorously rule out CMB. First, Harman's single-factor test was conducted: an exploratory factor analysis with all items constrained to a single factor explained 42.34% of the total variance, which is below the 50% critical threshold. Second, a full collinearity assessment was performed following Kock (2015), in which all constructs were regressed on a common factor. The resulting variance inflation factor (VIF) values ranged from 1.020 to 1.533, all below the 3.3 threshold (Table 2). Together, these results suggest that CMB is not a serious concern in this study.

**Table 2:** Full collinearity VIF assessment for common method bias.

Construct	Full collinearity VIF	Threshold	Assessment
AC	1.533	< 3.3	No CMB
INC	1.407	< 3.3	No CMB
IC	1.252	< 3.3	No CMB
Pre-AC	1.379	< 3.3	No CMB
Pre-INC	1.020	< 3.3	No CMB
Pre-IC	1.125	< 3.3	No CMB

Full collinearity VIF < 3.3 indicates absence of serious common method bias (Kock, 2015).

### 3.2 Measurement model assessment

The reflective measurement models were evaluated for reliability and validity following the guidelines for PLS-SEM (Hair et al., 2019). Table 3 presents the outer loadings for all indicators. Outer loadings for post-test constructs ranged from 0.746 to 0.880, with all values exceeding the 0.708 threshold recommended by Hair et al. (2019). Table 4 summarizes the construct reliability and convergent validity metrics. Internal consistency was robust (CR scores ranged from 0.808 to 0.890). Additionally, rho_A values ranged from 0.676 to 0.854. Although the rho_A values for Pre-AC (0.676) and IC (0.688) were slightly below the recommended threshold of 0.70, these values can be considered acceptable given the sensitivity of reliability estimates to constructs measured with fewer items (*k* = 3 and 2). Importantly, all CR values exceeded 0.80, which is the primary reliability criterion recommended for PLS-SEM (Hair et al., 2019). Furthermore, Cronbach's α is known to provide a lower-bound estimate of reliability for scales with few items (Sijtsma, 2009), and PLS-SEM scholars recommend prioritizing CR over α and rho_A when evaluating internal consistency (Hair et al., 2019). Therefore, the measurement model demonstrates acceptable reliability overall (Dijkstra and Henseler, 2015). Convergent validity was confirmed (AVE ranged from 0.589 to 0.803).

**Table 3:** Outer loadings of measurement items.

Item	AC	INC	IC	Pre-test loading
AC_1	**0.809**	0.330	0.206	0.399
AC_2	**0.774**	0.402	0.305	0.409
AC_3	**0.875**	0.456	0.255	0.433
INC_1	0.431	**0.836**	0.285	0.270
INC_2	0.307	**0.746**	0.374	0.202
INC_3	0.420	**0.843**	0.357	0.326
IC_1	0.262	0.382	**0.866**	0.170
IC_2	0.278	0.337	**0.880**	0.221

Bold values indicate loadings on assigned construct. All loadings > 0.708 (Hair et al., 2019).

**Table 4:** Construct reliability and convergent validity.

Construct	Items	Cronbach's α	rho_A	CR	AVE
AC	3	0.750	0.764	0.861	0.673
INC	3	0.740	0.760	0.850	0.655
IC	2	0.690	0.688	0.865	0.761
Pre-AC	3	0.660	0.676	0.821	0.605
Pre-INC	3	0.670	0.766	0.808	0.589
Pre-IC	2	0.760	0.854	0.890	0.803

CR, composite reliability; AVE, average variance extracted. CR > 0.70 and AVE > 0.50 are recommended thresholds (Hair et al., 2019). rho_A is the consistent reliability estimator for PLS-SEM (Dijkstra and Henseler, 2015).

Discriminant validity was assessed using both the Fornell-Larcker criterion (Table 5) and the HTMT ratio of correlations (Table 6). For the Fornell-Larcker criterion, the square root of each construct's AVE (diagonal values) was greater than its correlations with other constructs, confirming discriminant validity (Fornell and Larcker, 1981). All HTMT values were below the conservative threshold of 0.85 (Henseler et al., 2015), providing additional evidence of discriminant validity.

**Table 5:** Fornell–Larcker discriminant validity matrix.

Construct	AC	INC	IC	DTTM	Pre-AC	Pre-INC	Pre-IC
AC	**0.821**						
INC	0.484	**0.809**					
IC	0.310	0.411	**0.873**				
DTTM	0.092	0.009	−0.169	**1.000**			
Pre-AC	0.504	0.408	0.302	−0.099	**0.778**		
Pre-INC	0.316	0.335	0.116	−0.084	0.303	**0.768**	
Pre-IC	0.303	0.176	0.225	−0.076	0.237	0.257	**0.896**

Diagonal values (bold) represent the square root of AVE. Off-diagonal values represent inter-construct correlations. Discriminant validity is confirmed when diagonal values exceed all off-diagonal values in the same row and column (Fornell and Larcker, 1981).

**Table 6:** Heterotrait-Monotrait ratio of correlations.

Construct	AC	INC	IC
AC	—		
INC	0.636	—	
IC	0.432	0.587	—

All HTMT values < 0.85 confirm discriminant validity (Henseler et al., 2015).

### 3.3 Structural model assessment

Collinearity was assessed, with VIF values (1.010 to 1.129) indicating no multicollinearity issues. The model accounted for 27.4% of the variance in AC, 27.1% in INC, and 14.9% in IC. Bootstrapping with 5,000 resamples was executed.

Regarding direct effects, the DT intervention significantly enhanced AC compared to CTM (β = 0.287, *p* = 0.013, *f* ^2^ = 0.028), supporting H1. AC demonstrated a significant positive effect on both INC (β = 0.422, *p* < 0.001, *f* ^2^ = 0.216) and IC (β = 0.290, *p* < 0.001, *f* ^2^ = 0.088), supporting H2 and H3. The direct effect of DTTM on INC was non-significant (β = −0.026, *p* = 0.839), while the direct effect on IC was significantly negative (β = −0.373, *p* = 0.004).

Among the control variables, pre-test AC showed a strong significant positive effect on AC (β = 0.518, *p* < 0.001, *f* ^2^ = 0.366), and pre-test INC significantly predicted INC (β = 0.200, *p* = 0.001, *f* ^2^ = 0.049). However, pre-test IC did not significantly predict IC (β = 0.123, *p* = 0.120, *f* ^2^ = 0.016).

Mediation analysis using 5,000 bootstrap resamples revealed that AC fully mediated the relationship between the pedagogical intervention and INC (indirect effect β = 0.121, *p* = 0.022, 95% CI [0.031, 0.237]), with a non-significant direct effect, constituting indirect-only mediation (Zhao et al., 2010) and supporting H4. For IC, the indirect effect through AC was significant and positive (β = 0.083, *p* = 0.048, 95% CI [0.019, 0.188]), while the direct effect was significant and negative, constituting competitive mediation and supporting H5. Because this indirect effect was only marginally significant by the bootstrap t-statistic (*p* = 0.048) and IC was measured with two items, it should be interpreted with caution; the sensitivity analyses reported in Section 3.4 indicate that the competitive-mediation pattern was nonetheless robust to alternative IC operationalizations.

Predictive relevance was assessed via blindfolding (*d* = 7). *Q*^2^ values for AC (0.165) and INC (0.166) indicated medium predictive relevance, while IC (0.067) showed small predictive relevance, all exceeding zero and thus confirming the model's predictive capacity (Hair et al., 2019).

**Figure 2:**
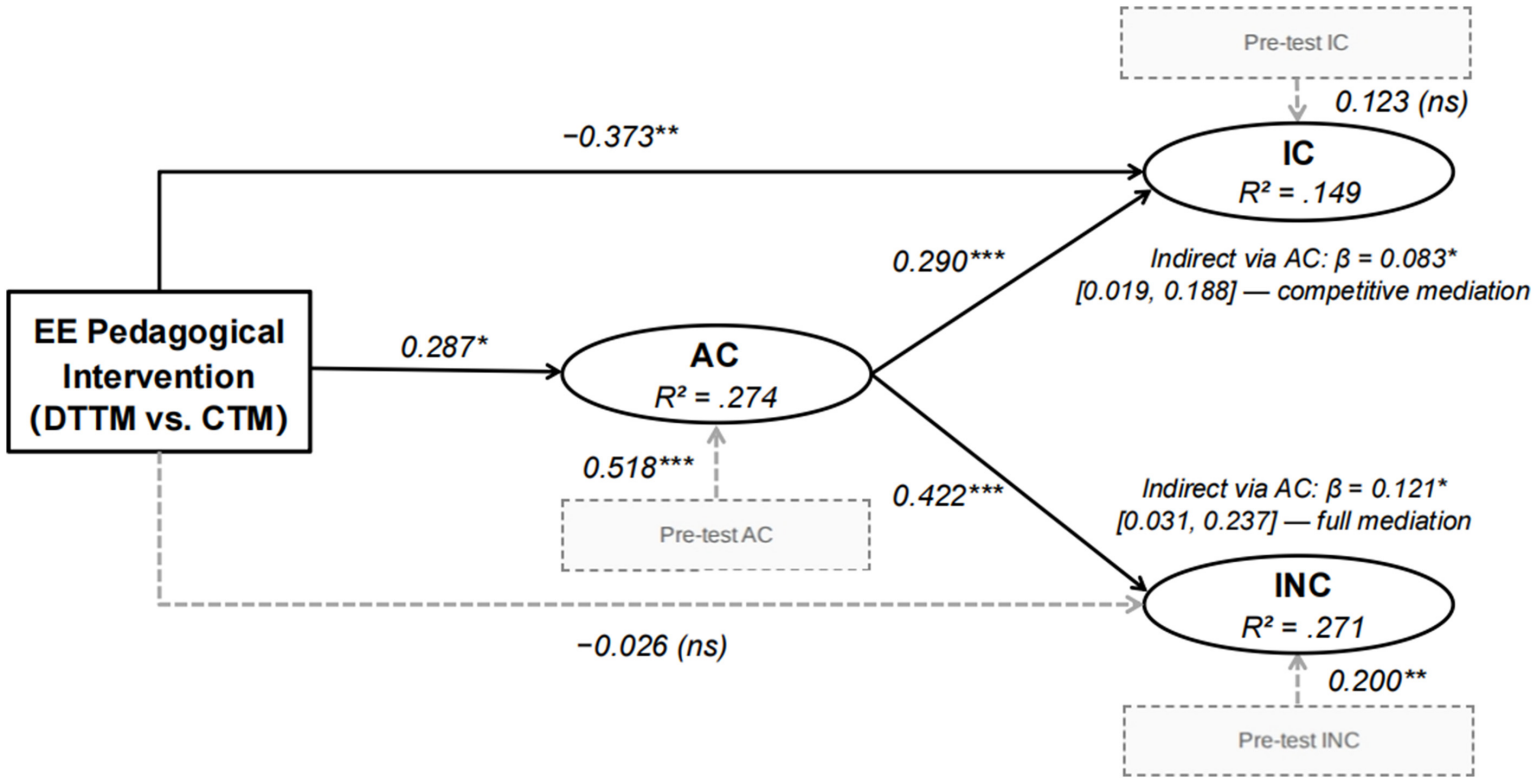
Structural model. Values are standardized PLS-SEM coefficients (5,000 bootstrap resamples); see Tables 7–9 for exact estimates and 95% CIs. EE, Entrepreneurship Education, CTM, Conventional Teaching Method, DTTM, Design Thinking Teaching Method, AC, Affective Attitude; INC, Innovation Competency; IC, Interpersonal Competency; Unless prefixed with “Pre-test,” AC, INC, and IC denote the post-test constructs; * *p* < 0.05, ** *p* < 0.01, *** *p* < 0.001.

The complete structural model results, including path coefficients, effect sizes (*f* ^2^), and mediation analyses with 95% bias-corrected bootstrap confidence intervals (CIs), are presented in Tables 7–9. The structural model with bootstrapping results is depicted in Figure 2.

**Table 7:** Structural model path coefficients and hypothesis testing results.

Path	β	*t*	*p*	*f* ^2^	95% CI [LL, UL]	Decision
Direct effects						
DTTM → AC (H1)	0.287	2.485	0.013 ^*^	0.028	[0.065, 0.521]	Supported
DTTM → INC	−0.026	0.204	0.839	0.000	[−0.269, 0.220]	n.s.
DTTM → IC	−0.373	2.897	0.004 ^**^	0.040	[−0.618, −0.119]	Sig. (Neg.)
AC → INC (H2)	0.422	6.651	< 0.001 ^***^	0.216	[0.289, 0.537]	Supported
AC → IC (H3)	0.290	4.064	< 0.001 ^***^	0.088	[0.144, 0.421]	Supported
Control paths						
Pre-AC → AC	0.518	8.137	< 0.001 ^***^	0.366	[0.372, 0.623]	Sig.
Pre-INC → INC	0.200	3.263	0.001 ^**^	0.049	[0.068, 0.311]	Sig.
Pre-IC → IC	0.123	1.554	0.120	0.016	[−0.049, 0.262]	n.s.

*f* ^2^ effect sizes: 0.02 = small, 0.15 = medium, 0.35 = large (Cohen, 1988). 95% CI, bias-corrected bootstrap confidence interval (5,000 resamples). ^*^ *p* < 0.05, ^**^ *p* < 0.01, ^***^ *p* < 0.001.

**Table 8:** Mediation analysis results with bootstrap confidence intervals.

Relationship	Effect type	β	*t*	*p*	95% CI [LL, UL]	Mediation type
**Innovation competency (INC)**						
DTTM → INC	Total	0.095	0.735	0.462	[−0.155, 0.353]	
DTTM → INC	Direct	−0.026	0.204	0.839	[−0.269, 0.220]	
**DTTM** ** → AC** ** → INC (H4)**	**Indirect**	**0.121**	**2.294**	**0.022** ^*^	**[0.031, 0.237]**	**Full (Indirect-only)**
Interpersonal competency (IC)						
DTTM → IC	Total	−0.290	2.170	0.030	[−0.554, −0.035]	
DTTM → IC	Direct	−0.373	2.897	0.004 ^**^	[−0.618, −0.119]	
**DTTM** ** → AC** ** → IC (H5)**	**Indirect**	**0.083**	**1.978**	**0.048** ^*^	**[0.019, 0.188]**	**Competitive**

Mediation classification follows Zhao et al. (2010): Full (indirect-only) mediation = significant indirect effect with non-significant direct effect; Competitive mediation = significant indirect and direct effects with opposite signs. Bold values indicate the hypothesized indirect (mediation) effects through AC (H4 and H5). ^*^p < 0.05, ^**^p < 0.01. A 95% CI that does not include zero indicates significance. Bootstrap resamples = 5,000.

**Table 9:** Effect sizes (*f* ^2^) and predictive relevance (*Q*^2^).

Endogenous construct	*R* ^2^	*Q* ^2^	Interpretation
AC	0.274	0.165	Moderate explanatory power; medium predictive relevance
INC	0.271	0.166	Moderate explanatory power; medium predictive relevance
IC	0.149	0.067	Weak explanatory power; small predictive relevance

*Q*^2^ obtained via blindfolding (omission distance *d* = 7). *Q*^2^ > 0 indicates predictive relevance; 0.02 = small, 0.15 = medium, 0.35 = large (Hair et al., 2019).

### 3.4 Sensitivity analyses for the two-item IC measure

Because IC was operationalized with two items, we assessed the robustness of the IC-related findings in three ways (Table 10). First, we re-estimated the full model twice, each time using a single IC item as the sole indicator of IC. The negative direct effect of the intervention on IC remained significant under both items (β = −0.346, *p* = 0.009; β = −0.313, *p* = 0.015), as did all other structural paths, and was independently corroborated by the non-parametric Quade's ANCOVA on observed scores (*F* = 8.38, *p* = 0.004). The positive indirect (buffering) effect via AC was stable in magnitude (β = 0.072 and β = 0.077); although its bootstrap t-statistic was marginal under one item (*p* = 0.051), its 95% bias-corrected bootstrap confidence interval excluded zero under both specifications ([0.017, 0.165] and [0.017, 0.173]), consistent with the confidence-interval-based inference recommended for indirect effects in PLS-SEM (Hair et al., 2019). The competitive-mediation pattern was therefore robust to single-item operationalization. Second, a consistent PLS (PLSc) re-estimation that corrects for measurement-error attenuation returned an inadmissible solution (bootstrapped *R*^2^ values and outer loadings exceeding 1.0 and undefined bootstrap standard errors for most paths), a recognized limitation of PLSc for two-indicator reflective constructs at moderate sample sizes (Sarstedt et al., 2016) we therefore do not interpret it. Across all admissible analyses, the negative direct effect on IC was fully robust, whereas the non-significant pre-post IC path is interpreted with caution, because a two-item (or single-item) measure does not adequately model measurement error and attenuates test-retest associations.

**Table 10:** Sensitivity analysis of the structural model across alternative IC operationalizations.

Path/effect	Two-item IC (main model)	Single-item IC (E1)	Single-item IC (E2)
Direct structural paths (standardized β)			
DTTM → AC	0.287^*^	0.289^*^	0.289^*^
DTTM → INC	−0.026	−0.027	−0.027
DTTM → IC	−0.373^**^	−0.346^**^	−0.313^*^
AC → INC	0.422^***^	0.426^***^	0.425^***^
AC → IC	0.290^***^	0.249^***^	0.268^***^
Pre-test AC → AC	0.518^***^	0.519^***^	0.519^***^
Pre-test INC → INC	0.200^**^	0.199^**^	0.200^**^
Pre-test IC → IC	0.123	0.117	0.106
Specific indirect effects (β [95% bias-corrected CI])			
DTTM → AC → INC	0.121 [0.031, 0.237]	0.123 [0.032, 0.238]	0.123 [0.032, 0.237]
DTTM → AC → IC	0.083 [0.019, 0.188]	0.072 [0.017, 0.165]	0.077 [0.017, 0.173]
Coefficient of determination (R^2^)			
AC	0.274	0.275	0.275
INC	0.271	0.274	0.273
IC	0.149	0.117	0.117

*N* = 198. E1 and E2 are the two interpersonal-competency items, each used in turn as the sole indicator of IC. ^*^ *p* < 0.05. ^**^ *p* < 0.01. ^***^ *p* < 0.001 (two-tailed).

## 4 Discussion

This study moved beyond the pervasive “black-box” assessments in EE by utilizing PLS-SEM to illuminate how DT is associated with competency development. Drawing on the CVT (Pekrun, 2006) as the primary theoretical lens, and supplemented by insights from dual-process theory (Gladwin and Figner, 2014) and social learning theory (Bandura, 1991), the findings are broadly consistent with the central proposition: DT may function as an “Affective Catalyst” by creating learning conditions that are theoretically consistent with enhanced control and value appraisals, thereby supporting the emotional engagement that accompanies complex competency acquisition. We emphasize at the outset that we use CVT as an interpretive framework rather than as a model that we test in full. Because control and value appraisals were not directly measured, the appraisal to emotion link is assumed on theoretical grounds, and our evidence speaks mainly to the emotion to competency segment of the chain. The interpretations below should therefore be read as consistent with CVT rather than as a direct confirmation of its appraisal mechanisms. Below, we discuss the key findings in relation to our theoretical framework, followed by practical implications and limitations.

### 4.1 The active learning paradox and the limitation of outcome-based comparisons

Our preliminary analyses revealed a pattern consistent with the “active learning paradox” documented in the broader pedagogical literature (Deslauriers et al., 2019): while DT significantly elevated students' emotional engagement (AC), it failed to show a direct, unmediated statistical superiority over traditional methods in hard competencies (INC and IC) when evaluated via mean scores. Deslauriers et al. (2019) empirically demonstrated that students in highly active classrooms often perceive themselves as learning less compared to students in passive, traditional lectures, primarily because the increased cognitive effort required in active learning is misinterpreted as poor learning.

This finding highlights a fundamental limitation of outcome-based comparisons in evaluating experiential pedagogies. Traditional methods often feature well-structured lectures that create a sense of learning fluency without necessarily fostering deep competency development (Deslauriers et al., 2019). If researchers rely solely on conventional mean-comparison metrics, they risk reaching the erroneous conclusion that DT merely makes classes “fun” without advancing rigorous academic capabilities. However, PLS-SEM reveals the true structural pathways: DT achieves competency parity (and ultimately may yield superiority in practical application) by shifting the underlying motivational mechanism to intrinsic emotional engagement through modified control and value appraisals (Pekrun, 2006). This result underscores the importance of employing process-oriented analytical approaches, such as mediation modeling, alongside traditional outcome measures when evaluating complex pedagogical interventions.

### 4.2 Full mediation of affective attitude on innovation competency

A central finding of this study is the full (indirect-only) mediation of AC on the relationship between DT and INC. The direct effect of the DT intervention on INC was entirely non-significant (β = −0.026, *p* = 0.839, 95% CI [−0.269, 0.220]), while the indirect effect through AC was both statistically significant and practically meaningful (β = 0.121, *p* = 0.022, 95% CI [0.031, 0.237]). According to the mediation typology of Zhao et al. (2010), this constitutes indirect-only mediation, suggesting that, in this sample, AC is the primary pathway through which DT relates to INC.

This finding can be interpreted through two complementary theoretical perspectives. From a CVT standpoint, DT's pedagogical structure, particularly its emphasis on normalizing failure through rapid prototyping (which may enhancing perceived control) and connecting tasks to real-world user needs (which may enhancing intrinsic value), creates the appraisal conditions that CVT predicts will generate positive activating emotions such as enjoyment and engagement (Pekrun, 2006; Simonton and Garn, 2020). These emotions, in turn, facilitate the cognitive flexibility, risk tolerance, and divergent thinking required for innovation (Pekrun, 2006). From a dual-process perspective (Gladwin and Figner, 2014; Tinajero et al., 2024), innovation is fundamentally a “hot cognition” process, involving decision-making under conditions of high uncertainty and risk, that cannot be effectively executed in a purely “cold,” de-emotionalized state. DT teaching tools such as brainstorming, rapid prototyping, and iterative feedback are essentially methodological guidelines operating at the “cold cognition” level (Ovbiagbonhia et al., 2019). These two perspectives converge on a shared implication: affective activation may be an important enabling condition translating DT's methodological tools into actual innovation performance.

This interpretation is particularly relevant to the Chinese educational context. For students accustomed to standardized assessments and high-stakes evaluation, years of conditioning in failure-avoidant environments may have produced deeply ingrained avoidance patterns against uncertainty (Fang et al., 2023). In such contexts, merely providing DT's “cold cognition” tools may be insufficient to bypass these psychological barriers. The non-significant direct effect of DT on INC (β = −0.026, *p* = 0.839) should therefore not be interpreted as evidence that DT tools are ineffective, but rather as indicating that these tools require affective activation to function as intended. By creating conditions theoretically consistent with enhanced control and value appraisals, specifically by normalizing failure through low-fidelity prototyping (which may increasing perceived control) and focusing on real-world human needs (which may increasing intrinsic value)—DT fosters a positive AC. It is only after this affective engagement is established that students experience the psychological safety required to engage in innovative thinking. Consistent with this reasoning, Li (2026) demonstrated that uncertainty-enhanced inquiry tasks improved both students' control beliefs and their learning enjoyment, supporting the notion that pedagogical designs modifying appraisal conditions can effectively generate positive achievement emotions.

### 4.3 Competitive mediation on interpersonal competency

A more tentative and exploratory pattern emerged in the pathway for IC. The data exhibited a highly significant negative direct effect of the DT intervention on IC (β = −0.373, *p* = 0.004, 95% CI [−0.618, −0.119]), alongside a significant positive indirect effect via AC (β = 0.083, *p* = 0.048, 95% CI [0.019, 0.188]). In the mediation framework of Zhao et al. (2010), this pattern, in which direct and indirect paths are both significant but possess opposite signs, constitutes competitive mediation.

The negative direct effect on IC has several possible explanations, and we give priority to process-level mechanisms that do not depend on cultural assumptions. First, intensive peer critique generates task and interpersonal conflict, and meta-analytic evidence indicates that such conflict tends to lower team member satisfaction and the quality of interpersonal experience independently of cultural setting (De Dreu and Weingart, 2003). Second, the move from structured instruction to unstructured collaboration is itself demanding and novel, and the broader active learning literature shows that such transitions can transiently depress students' subjective evaluations of their own performance even when objective learning improves (Deslauriers et al., 2019). Finally, because IC was assessed with only two items tapping relationship building, the negative effect may pertain to a narrow facet of IC rather than to the construct as a whole. A further possibility, which we did not test directly and treat as the most speculative of these accounts, is cultural, and we develop it in the following paragraph. These accounts are not mutually exclusive, and disentangling them will require studies that directly measure cultural orientation, conflict experience, and a broader set of interpersonal indicators.

The negative direct effect can be interpreted through CVT's four-quadrant model applied to the interpersonal domain. DT demands “radical collaboration,” forcing students into an unstructured environment where they must rapidly synthesize conflicting ideas and critique peers' prototypes. We propose that this pedagogical design creates a specific appraisal configuration within the interpersonal sub-domain: while DT enhances the overall value of the task through its empathy phase (Chen et al., 2020), radical collaboration may simultaneously reduce students' perceived control in the interpersonal domain. In the Chinese educational context, where interpersonal harmony and face concern (mianzi) are deeply valued (King and Wei, 2018), students may be particularly sensitive to the unpredictability of candid peer criticism. They cannot anticipate how direct feedback will affect peer relationships, nor can they manage group conflicts using their habitual harmony-preservation strategies. According to CVT, the combination of high value and low control in a specific domain reliably generates anxiety and threat-related emotions (Heckhausen et al., 2010; Pekrun, 2006), which in turn trigger defensive behaviors such as avoidance, rigidity, and social withdrawal (Vu et al., 2021). This mechanism offers one plausible account of the negative direct effect.

It is important to note, however, that this interpretation incorporates culturally specific assumptions about face concern and harmony norms that were not directly measured in the present study. While the reasoning is consistent with both CVT's predictions and the existing literature on Chinese collectivist culture (Hofstede, 2001; King and Wei, 2018), it should be regarded as a plausible *post-hoc* explanation rather than a confirmed causal account. Future research should directly measure cultural variables such as face concern and conflict avoidance tendencies to test whether the competitive mediation pattern is indeed culturally contingent.

The positive indirect effect through AC can be understood through CVT's emotion-regulation mechanisms. When DT's overall pedagogical design successfully fosters a strong AC, meaning that students' global emotional engagement with the entrepreneurial learning experience is positive despite domain-specific interpersonal anxiety, this positive affect may facilitate cognitive reappraisal processes. Students experiencing high overall AC may engage in cognitive reappraisal (Balzarotti et al., 2017), reinterpreting interpersonal friction as constructive feedback rather than a personal threat. Research on emotion regulation has established that cognitive reappraisal is an effective strategy for reducing negative emotions and enhancing adaptive functioning in stressful interpersonal situations (Balzarotti et al., 2017). Under this reappraisal, interpersonal conflict no longer triggers an anxiety-avoidance cycle but may become productive because it brings clear feedback and diverse perspectives. Additionally, from a social learning perspective (Bandura, 1991), the positive affective state creates the psychological conditions (i.e., openness, reduced defensiveness, and willingness to engage) under which students can effectively observe, model, and internalize constructive collaborative behaviors demonstrated during the DT process.

Furthermore, examining the control variables reveals that pre-test IC had no significant predictive power over IC (β = 0.123, *p* = 0.120, 95% CI [−0.049, 0.262]). This finding is noteworthy but requires cautious interpretation. We consider several possible explanations. First, the IC scale comprised only two items, which may contribute to measurement instability across time points and attenuate the pre-post correlation. Second, natural fluctuations in self-reported IC over a 12-week period cannot be ruled out. Third, the pattern is also consistent with CVT's situational dependency principle, under which achievement emotions and associated competencies are dynamic states that may be recalibrated when learners enter fundamentally new situations (Forsblom et al., 2021; Pekrun, 2024); on this reading, DT's novel collaborative nature may renders students' prior interpersonal strategies less applicable, so that subsequent IC development depends more on current affective engagement than on prior standing, consistent with evidence that novel contexts can reset interest trajectories (Hidi and Renninger, 2006). However, because a two-item (or single-item) measure attenuates test-retest associations, and the consistent PLS estimator that could correct for this returned an inadmissible solution (Section 3.4), we cannot distinguish a genuine reset of prior standing from measurement-induced attenuation. We therefore do not advance a “Reset Effect” as a finding, and leave this question to future research employing multi-item measures and multiple measurement waves.

### 4.4 Practical implications

#### 4.4.1 Implications for universities and policymakers

At the institutional level, the finding that competency acquisition is substantially mediated by affective engagement has direct implications for how universities evaluate EE programs. Evaluating EE solely through end-of-semester standardized exams or polished business plans may systematically underestimate the developmental value of experiential approaches such as DT, which operate primarily through affective rather than direct cognitive pathways. Universities should consider incorporating process-oriented assessment metrics that capture students' emotional engagement, iterative learning behaviors, and reflective practices alongside conventional outcome measures.

Institutions should provide funding, dedicated physical maker-spaces, and academic credit structures that reward process-oriented learning, trial-and-error, and active community engagement. Additionally, the competitive mediation finding for IC suggests that universities implementing DT programs should provide structured support for students navigating the transition from conventional to radical collaboration. This might include pre-program workshops on constructive feedback, conflict resolution training, or facilitated reflection sessions that help students process the emotional challenges of collaborative innovation (King and Wei, 2018). Such scaffolding could mitigate the negative direct effect of interpersonal friction while preserving the positive indirect pathway through affective engagement.

#### 4.4.2 Implications for teachers and educators

For front-line educators, the findings support a paradigm shift from a “skills-first” logic to an “Affective-First” instructional sequencing. Because hard competencies (INC and IC) are fully or competitively mediated by affective engagement, teachers should consider prioritizing emotional activation in the initial weeks of a course. Drawing on CVT's appraisal logic, this means designing early learning experiences that maximize both perceived control (e.g., low-stakes, failure-tolerant activities) and perceived value (e.g., connecting tasks to real-world impact), thereby creating the appraisal conditions most conducive to positive activating emotions (Pekrun, 2006; Simonton and Garn, 2020).

Educators must also be acutely aware of the “active learning paradox” and the cultural constraints of mianzi. To prevent the negative direct effects of cognitive friction from overwhelming students' interpersonal experience, teachers must act as empathetic coaches rather than traditional lecturers. They should explicitly validate student frustration, provide robust psychological safety during peer critique, and continually reinforce the intrinsic value of the design tasks to sustain affective engagement. Specific strategies might include: (a) beginning courses with low-stakes, high-engagement activities that build confidence; (b) explicitly teaching and modeling constructive feedback language that separates ideas from personal identity; (c) creating structured opportunities for students to reflect on the value of their projects; and (d) providing just-in-time emotional support when students encounter the inevitable frustrations of iterative design.

#### 4.4.3 Implications for students

For students, particularly those accustomed to the structured, high-pressure environments of the Chinese education system, maximizing the benefits of DT requires a conscious cognitive shift. Students should recognize that the confusion and interpersonal friction experienced during radical collaboration are not indicators of failure, but essential components of the innovation process. By developing metacognitive awareness of their own appraisal processes (as described by CVT), students can learn to recognize when their perceived control is being threatened (e.g., during peer critique) and actively work to maintain their value appraisals (e.g., by reconnecting with the user need they are addressing). This metacognitive capacity can help students navigate the emotional landscape of DT more effectively, ultimately enhancing both their affective engagement and competency development.

### 4.5 Limitations and future research

While this study offers valuable insights into the affective mechanisms underlying DT's effects on entrepreneurial competency development, several limitations should be considered when interpreting the findings.

First, the sample was drawn from a single Chinese university, limiting cross-cultural generalizability. The sample was also highly homogeneous, predominantly female (73.7%) and almost entirely sophomores (98.5%), which further limits generalizability across gender, year level, and institutional context; findings should be extended to male students, other year levels, and other institutional settings only with caution. The cultural interpretation of the negative direct effect on IC (Section 4.3) incorporates assumptions about face concern and collectivism that, while theoretically grounded (King and Wei, 2018), were not directly measured. Future research should conduct multi-group analyses comparing Western individualistic cohorts with Eastern collectivist samples, incorporating validated cultural measures (e.g., Ting-Toomey's face negotiation scales) as potential moderators of the competitive mediation pattern.

Second, all variables were assessed through self-report measures at a single post-test time point, raising concerns about CMB. Although our CMB analyses (Section 3.1.1) did not reveal serious concerns, and procedural remedies were implemented (anonymous administration, item randomization; Podsakoff et al., 2003), future research should incorporate objective or multi-source competency measures, such as instructor ratings, peer evaluations, or behavioral assessments of prototype quality, to provide a more robust test of the proposed pathways.

Third, the IC scale comprised only two items, which limits content coverage and may have contributed to the lower reliability observed at post-test (α = 0.69) as well as the non-significant pre-post path for IC. The sensitivity analyses in Section 3.4 indicate that, despite this limitation, the negative direct effect on IC was robust across single-item operationalizations, whereas the non-significant pre-post path could not be distinguished from measurement-induced attenuation and is therefore not interpreted substantively. Future studies should develop or adopt more comprehensive IC measures with at least three to four items to improve measurement precision and test-retest stability. More broadly, because the two items tap mainly relationship building rather than the fuller interpersonal domain such as conflict management and communication, the negative direct effect should be read as pertaining to a narrow facet rather than to IC as a whole, and the low reliability of the measure likely attenuates and destabilizes the already marginal indirect effect. We therefore regard the direction of the competitive mediation pattern as robust but its magnitude and precision as provisional, and we treat the IC findings as more tentative than the three-item INC findings.

Fourth, the within-instructor design, while effectively controlling for instructor-related confounds, may introduce experimenter expectancy effects (Rosenthal, 2002). The instructor may have unconsciously invested differential enthusiasm across conditions. Future research should employ multi-instructor designs or incorporate blind observer evaluations to mitigate this threat. Relatedly, because participants were not randomly assigned and intact classes were used, unmeasured pre-existing differences between the groups cannot be excluded as alternative contributors to the observed effects, and causal claims should therefore be made cautiously.

Fifth, as acknowledged in Section 1.2, the operationalization of AC as the affective component of students' attitudes toward EE represents a broader construct than the discrete achievement emotions specified by CVT. While we have argued that AC serves as a meaningful aggregate indicator of cumulative appraisal-emotion episodes (Goetz et al., 2006; Pekrun and Stephens, 2010), future research should incorporate direct measures of CVT's appraisal dimensions (control and value) and specific achievement emotions (e.g., using the Achievement Emotions Questionnaire; Pekrun et al., 2011) to provide a more complete test of the CVT mechanism. Such research would enable testing of the full appraisal → emotion → competency chain that the current design can only partially examine.

Sixth, relying on pre/post cross-sectional measurements captures the net affective outcome but misses transient emotional fluctuations. Future studies could employ the Experience Sampling Method to track real-time emotional volatility (e.g., frustration spikes during prototype failure) to map the micro-dynamics of the appraisal-emotion-competency process. Additionally, the *R*^2^ for IC (14.9%) indicates that a substantial proportion of variance remains unexplained, suggesting that additional variables (e.g., self-efficacy, personality traits, prior entrepreneurial exposure) should be incorporated in future models to provide a more comprehensive account of IC development.

Finally, future research should explore the specific components of DT that most effectively modify control and value appraisals. While this study treated DT as a unified intervention, different elements (empathy work, prototyping, iteration) may have distinct effects on students' appraisal processes. Fine-grained analysis of these components could inform more targeted and effective pedagogical designs.

## 5 Conclusions

As higher education seeks to cultivate students' capacity to navigate uncertainty and innovation challenges, this study contributes to understanding the psychological processes associated with DT-based EE. By applying CVT as an interpretive lens rather than testing the full appraisal to emotion to outcome sequence, this study suggests that affective engagement may represent an important pathway connecting DT pedagogy with entrepreneurial competency-related outcomes.

The findings suggest that DT is not only a set of cognitive problem-solving techniques but also a learning approach that may create conditions consistent with enhanced perceived control, task value, and emotional engagement. AC emerged as a full mediator between DT and INC, indicating that emotional engagement may play an important role in supporting the cognitive flexibility and risk tolerance required for innovative learning processes.

An exploratory competitive mediation pattern was also observed for IC. While emotional engagement appeared to buffer the negative association between DT and IC outcomes, this finding should be interpreted cautiously because IC was assessed using only two items and cultural orientations were not directly measured. Future research incorporating comprehensive IC measures and direct assessments of cultural and appraisal-related variables is needed to clarify this relationship.

Overall, the findings suggest that EE should not evaluate experiential pedagogies solely through final competency outcomes. Instead, educators should consider the affective processes through which students engage with challenging, uncertain, and collaborative learning experiences. Future longitudinal and multi-method studies are encouraged to further examine the dynamic appraisal to emotion to competency processes proposed by CVT.

## Data Availability

The raw data supporting the conclusions of this article will be made available by the authors, without undue reservation.
